# Root morphology and seed and leaf ionomic traits in a *Brassica napus* L. diversity panel show wide phenotypic variation and are characteristic of crop habit

**DOI:** 10.1186/s12870-016-0902-5

**Published:** 2016-10-04

**Authors:** C. L. Thomas, T. D. Alcock, N. S. Graham, R. Hayden, S. Matterson, L. Wilson, S. D. Young, L. X. Dupuy, P. J. White, J. P. Hammond, J. M. C. Danku, D. E. Salt, A. Sweeney, I. Bancroft, M. R. Broadley

**Affiliations:** 1 School of Biosciences, University of Nottingham, Sutton Bonington Campus, Loughborough, LE12 5RD UK; 2 Ecological Sciences, The James Hutton Institute, Invergowrie, Dundee, DD2 5DA UK; 3 Distinguished Scientist Fellowship Program, King Saud University, Riyadh, 11451 Kingdom of Saudi Arabia; 4 School of Agriculture, Policy and Development and the Centre for Food Security, University of Reading, Whiteknights, PO Box 237, Reading, RG6 6AR UK; 5 University of Aberdeen, Institute of Biological and Environmental Sciences, Cruickshank Building, St Machar Drive, Aberdeen, AB24 3UU UK; 6 Department of Biology, University of York, Heslington, York, YO10 5DD UK

**Keywords:** Canola, Ionomics, Mineral concentration, High-throughput phenotyping, Root morphology, Seed size, Leaf/seed elemental ratios

## Abstract

**Background:**

Mineral nutrient uptake and utilisation by plants are controlled by many traits relating to root morphology, ion transport, sequestration and translocation. The aims of this study were to determine the phenotypic diversity in root morphology and leaf and seed mineral composition of a polyploid crop species, *Brassica napus* L., and how these traits relate to crop habit. Traits were quantified in a diversity panel of up to 387 genotypes: 163 winter, 127 spring, and seven semiwinter oilseed rape (OSR) habits, 35 swede, 15 winter fodder, and 40 exotic/unspecified habits. Root traits of 14 d old seedlings were measured in a ‘pouch and wick’ system (*n* = ~24 replicates per genotype). The mineral composition of 3–6 rosette-stage leaves, and mature seeds, was determined on compost-grown plants from a designed experiment (*n* = 5) by inductively coupled plasma-mass spectrometry (ICP-MS).

**Results:**

Seed size explained a large proportion of the variation in root length. Winter OSR and fodder habits had longer primary and lateral roots than spring OSR habits, with generally lower mineral concentrations. A comparison of the ratios of elements in leaf and seed parts revealed differences in translocation processes between crop habits, including those likely to be associated with crop-selection for OSR seeds with lower sulphur-containing glucosinolates. Combining root, leaf and seed traits in a discriminant analysis provided the most accurate characterisation of crop habit, illustrating the interdependence of plant tissues.

**Conclusions:**

High-throughput morphological and composition phenotyping reveals complex interrelationships between mineral acquisition and accumulation linked to genetic control within and between crop types (habits) in *B. napus*. Despite its recent genetic ancestry (<10 ky), root morphology, and leaf and seed composition traits could potentially be used in crop improvement, if suitable markers can be identified and if these correspond with suitable agronomy and quality traits.

**Electronic supplementary material:**

The online version of this article (doi:10.1186/s12870-016-0902-5) contains supplementary material, which is available to authorized users.

## Background

Plants require at least 14 essential mineral elements to complete their life-cycles [1]. These include nitrogen (N), phosphorus (P), potassium (K), calcium (Ca), magnesium (Mg) and sulphur (S), which are macronutrients required in large amounts (typically 1000– > 10,000 mg kg^−1^ leaf dry weight, DW). The micronutrients chlorine (Cl), boron (B), iron (Fe), manganese (Mn), zinc (Zn), copper (Cu), nickel (Ni) and molybdenum (Mo) are required in smaller amounts (typically 0.1–100 mg kg^−1^ leaf DW) [2]. Plants also accumulate non-essential elements, some of which have little or no effect on plant growth and development at the concentrations they occur in nature, and others of which may have beneficial and/or detrimental effects depending upon their concentrations in plant tissues. These include arsenic (As), cadmium (Cd), selenium (Se), silicon (Si) and sodium (Na).

Most mineral elements are taken up in ionic form from the soil solution by plant roots. Traits/phenes affecting root morphology and anatomy play a key role in the acquisition of mineral nutrients by plants and impact on crop yields [3–5]. For example, increased root hairs and shallower basal root growth angles can increase P uptake [6, 7]. Reduced allocation of carbon to root structures via increased aerenchyma and reduced cortical cell file formations [8] and smaller root diameter [9] may allow some plants more efficient access to larger soil volumes, and thereby water and nutrients. The subsequent uptake and utilisation of mineral elements by plants is controlled by traits affecting ion transport, translocation and sequestration [1]. Mineral elements in both chelated and free-ionic forms move across the root via apoplastic (extracellular) and symplastic (intracellular) pathways to the stele. Following xylem loading and subsequent transport to transpiring leaf tissues, elements are taken up from the leaf apoplast by specific cell types. Translocation of mineral elements in the plants to non-transpiring or xylem-deficient tissues occurs via the phloem [10, 11]. Some elements are highly mobile in phloem tissues (K, Na, Mg, Cd, N, P, S, Se and Cl), some are relatively immobile in the phloem (Ca and Mn), and some elements have intermediate mobility (B, Fe, Zn, Cu, Mo and I) [10–12].

The term ‘ionome’ defines the complement of mineral elements in all of their chemical forms within an organism or tissue, irrespective of whether they are essential or non-essential [13]. The ionome is thus the inorganic subset of the metabolome at a given moment in space and time, which varies at all scales. Within an individual plant, an ionome is specific to tissue type and developmental stage; e.g. seed, fruit and tuber concentrations of Ca are lower than leaf concentrations of Ca due to its limited phloem mobility [14, 15]. Between individuals, the ionome of a specific tissue type varies due to environmental and genetic factors at all scales and this can be observed as differences between populations, species, and plant families [13, 14, 16–18].

Variation in the ionomes of edible crop tissues has enabled identification of quantitative trait loci (QTL) linked to mineral composition and important to human and animal nutrition in several crop species [3, 19, 20]. For example, genetic loci affecting the mineral composition of leaves of *Brassica oleracea* [21], *Brassica rapa* [22], *Brassica napus* [23] and *Lotus japonicus* [24] have been identified. In the study of Bus et al. [23], there were strong pair-wise positive correlations in the shoot concentrations of many of the 11 mineral elements in 30 d old *B. napus* (>500 genotypes). Furthermore, there were many pair-wise negative correlations between the shoot concentrations of several elements, notably Ca and K, and numerous leaf and seedling size related traits. Plant ionomes are also amenable to genetic dissection using natural and induced genetic variation via mutagenesis, using association mapping and reverse genetic approaches. Several genes underlying variation in mineral nutrient acquisition and translocation have recently been identified. For example, in *Arabidopsis thaliana*, a deletion mutant with a reduced leaf Ca concentration led subsequently to the identification of *ESB1* (*Enhanced Suberin Biosynthesis 1*) which affects Casparian Band formation [25, 26]. A mutant with reduced leaf Mg, Ca, Fe, and Mo and increased leaf Na and K concentration was similarly associated with reduced sphingolipid biosynthesis [27]. A variety of other *Arabidopsis* genes are associated with phenotypic variation in leaf As [28], Cd [29], K [30], S and Se [31].

*Brassica napus* is an important crop in global terms, with crop types including oilseed rape (OSR), vegetable swede, and fodder crops. Currently, oilseed types of OSR are the third largest source of vegetable oil globally after soybean and oil palm. Worldwide production of OSR was 72.8 Mt in 2013 [32]. Other uses for OSR oils include biodiesel and rape meal for animal feeds, and co-products, including vitamin E (tocopherol) and cholesterol lowering compounds (phytosterols) from the oil, and waxes from pod walls with medical/cosmetic properties. Further industrial oils are currently underexploited but could increase economic margins for farmers. There is considerable scope for improvement of yield of seeds and co-products if suitable traits can be identified and introduced into well-adapted varieties, for example, through improvements in yield and resource-use efficiency [33, 34]. Worldwide average yields for OSR have increased from 1.5 to 2 t ha^−1^ from 2000 to 2013. Yields are higher in Western Europe, with 2013 average yields of 3.5 t ha^−1^. The long term average yield of UK OSR is 3.1 t ha^−1^ [35], which is much less than UK wheat (8.1 t ha^−1^) and UK barley (6.4 t ha^−1^) yet it is similarly nutrient-intensive [36]. The yields of UK OSR are also far less than their estimated potential of >6.5 t ha^−1^ [35].

The aim of this study was to determine the phenotypic diversity in root morphology, shoot ionomic (leaf and seed) and seed size/yield traits within a broad genetic diversity panel of *B. napus* (encompassing all crop types) and to identify their relationship to crop habit. Determining the phenotypic diversity in these traits, and their interrelationships, in this population could inform subsequent studies to dissect the genetic bases and identify markers in traits relevant for crop improvement [37]. An increased understanding of these traits could also help in breeding strategies via more conventional means. To our knowledge, no previous studies have simultaneously characterised the phenotypic variation in root morphology, ionomes and seed size from such a large diversity panel, which is likely to capture most of the species-wide variation in these traits in *B. napus*.

## Methods

### Plant material for all experiments

Inbred lines of *Brassica napus* L. genotypes were used in this study. These were from the ERANET-ASSYST consortium diversity population [23, 38–40]. A core panel of 387 genotypes were selected, comprising 163 winter, 127 spring, and seven semiwinter oilseed rape (OSR), 35 swede, 15 winter fodder, and 40 exotic/unspecified habits (Additional file 1: Table S1). Two cultivation systems were deployed. Seedling root traits were determined in a ‘pouch and wick’ hydroponic system in a controlled environment (CE) room. Leaf and seed mineral composition traits were measured on compost-grown plants grown in a designed experiment in a polytunnel.

### Root phenotyping in a pouch and wick system

The ‘pouch and wick’ high-throughput phenotyping (HTP) system was reported previously [5, 41]. This system comprised growth pouches assembled from blue germination paper (SD7640; Anchor Paper Company, St Paul, MN, USA), re-cut to 24 × 30 cm and overlain with black polythene (Cransford Polythene Ltd, Woodbridge, UK). Along one of the shorter edges, the paper and polythene were clipped together to an acrylic rod (Acrylic Online, Hull, UK) using ‘bulldog’-type fold-back clips. The growth pouches were suspended above plastic drip trays, supported within lightweight aluminium/polycarbonate frames (KJN Aluminium Profiles, Leicester, UK). Each drip tray contained 2 L of 25 % strength Hoagland’s solution (No. 2 Basal Salt Mixture, Sigma Aldrich, Dorset, UK) made with deionised water. Drip trays were replenished with 500 mL of deionised water every 3 d. Prior to sowing, the pouches were suspended above the nutrient solution for a minimum of 4 h to become fully saturated. Within each aluminium frame, nine drip trays were used, arranged in three columns and three rows. Pouches were allocated randomly to drip trays, 10 or 11 pouches per drip tray, thus 96 pouches and 192 plants per frame (i.e. a single plant on each side of the paper). A total of four frames were used in each experimental run, giving a potential sample size of 768 plants per run within the CE room. The CE room was 2.2 m width, 3.3 m length, 3.0 m height, set to a 12 h photoperiod 18/15 °C day/night temperatures and relative humidity of 60–80 %. Photosynthetically Active Radiation (PAR; measured at plant height with a 190 SB quantum sensor; LI-COR Inc., Lincoln, NE, USA) was approximately 207 μmol m^−2^ s^−1^, generated by 400 W white fluorescent lamps (HIT 400w/u/Euro/4 K, Venture Lighting, Rickmansworth, UK).

A single seed was sown on each germination paper, in the middle of the upper edge of the paper, by pressing the seed into the paper. The potential effect of seed size on root traits was controlled for by selecting individual seeds which spanned a range of sizes for each genotype, therefore giving a mean seed diameter of ~1.8 mm for each genotype. Seeds of each genotype were sieved using mesh with a diameter (Ø) of 1.4, 1.7, 2.0 and 2.36 mm (Scientific Laboratory Supplies Ltd, Hessle, UK). Seed retained within the mesh of each faction were selected such that 25 % of seed represented each Ø-category for each genotype. Where insufficient seeds were available for a given Ø-category, the next smallest Ø-category was used instead.

Fourteen days after sowing (DAS), the polythene sheets were removed from all pouches and images were taken of the germination paper and root system for downstream image analysis. Images were taken using a Digital Single Lens Reflex (DSLR) camera (Canon EOS 1100D, Canon Inc., Tokyo, Japan) with a focal length of 35 mm at a fixed height of 75 cm. The root images from the HTP system were renamed with each sample’s unique experimental design information using Bulk Rename Utility (Version 2.7.1.3, TGRMN Software, www.bulkrenameutility.co.uk). Images were cropped by reducing extraneous pixels on bulked images, using XnConvert (Version 1.66, www.xnconvert.com). Cropped images were analysed using RootReader2D (RR2D) [42]. First, a ‘batch process’ was carried out which automatically ‘thresholds’, ‘skeletonises’ and ‘builds segments’ of all images in bulk. The root system was then measured on individual images by placing a marker at the base and tip of the primary root. From these markers, RR2D automatically calculates primary root length (PRL), lateral root length (LRL) of all laterals, and lateral root number (LRN). Further traits calculated from these data included total root length (TRL = PRL + LRL), mean lateral root length (MLRL = LRL/LRN) and lateral root density (LRD = LRN/PRL). A database was developed which integrated the experimental design information from the image name, with the RR2D measurements for each sample, using a programming script (2.7.10; Python Software Foundation, www.python.org).

Of the core panel of 387 genotypes, 354 genotypes comprising 156 winter, 124 spring and seven semiwinter OSR, 14 winter fodder, 33 swede and 20 exotic/unspecified types were screened. Two additional reference winter OSR lines were screened in each experimental run. Each experimental run comprised 32 genotypes, of 24 individuals per genotype. There were 16 experimental runs in total. This equates to a total of 11,176 potential images. An image was removed from analysis if the seed had failed to germinate, or if the seed had rolled down the paper and therefore the shoot failed to emerge above the pouch, or if the seedling was stunted with a radicle < 3 cm, or the radicle appeared deformed such as being twisted around the seed. Overall, 29 % of samples were removed from analysis; excluded data are noted in Additional file 1: Table S2 and all images are available on request.

The relative contribution of genotypic and non-genotypic variance components underlying variation in root traits were calculated using a REML (REsidual Maximum Likelihood) procedure according to the model [(run/frame/column/tray/paper-side) + habit + seed size + genotype]. Genotype was subsequently added as a fixed factor to estimate genotype-means of root traits.

### Leaf and seed mineral composition traits in soil-grown plants

#### Growth of plant material

Seed of all genotypes were sown directly into fine-grade (<3 mm particle size) compost-based growing media (Levington Seed & Modular + Sand -F2S; Everris Ltd., Ipswich, UK) in modular propagation trays (650 plants m^−2^; internal Ø 2.5 cm, module volume 55 cm^3^; Type ‘104’, Desch Plantpak, Essex, UK). Sowing took place from 22 to 29 October 2013. The compost was covered with perlite and transferred to a glasshouse vented at 15 °C (controlled by TomTech μClimate, Spalding, Lincs). Supplementary, artificial lighting (Philips Master GreenPower SON-T 400 W bulbs controlled by Grasslin Uni 45 timer) was used to maintain day lengths of 12 h light d^−1^. Watering was once daily by hand as required until transplantation. From 16 to 29 January 2014, five plants of each genotype were transplanted into individual 5 L pots (internal Ø 22.5 cm; height 18 cm) containing Levington C2 compost (Scotts Professional, Ipswich, UK). Pots were arranged within two single-skinned polytunnels (with a Visqueen Luminance Skin, Northern Polytunnels, Colne, UK) with no additional lighting or heating, at the Sutton Bonington Campus of the University of Nottingham (52°49'58.9" N, 1°14'59.2" W).

Pots were arranged in a randomised block design of five replicate blocks using an R script (personal communications, Edmondson RA, superseded [43]). Three replicates were allocated to one polytunnel, two to the other. Each replicate comprised 432 units, including one of each of the 387 core genotypes, plus 16 reference genotypes added to enable more accurate normalisation. A further 29 genotypes were included to fill gaps. Each replicate block was split into 12 sub-blocks of 36 genotypes, which were allocated at random. Where a lack of germination meant that insufficient plants were available at the transplanting stage, empty, compost-filled pots were used in their place.

Automatic irrigation was controlled in each polytunnel by a Hunter Irrigation Controller (Hunter Industries, San Marcos, CA, USA, provided by Hortech Systems Ltd., Holbeach, UK). Water from a header tank was distributed by a pump (DAB Active JI112M; DAB Pumps Ltd. Bishop's Stortford, UK) such that each pot received 133 mL of water at 08:00, 12:00 and 16:00 each day, via a low density polyethylene (LDPE) pipe based irrigation system fitted with compensated, non-leaking (CNL) drippers at 4 L h^−1^ capacity. Each CNL dripper supplied four pots using an attached, four-tipped manifold (Netafim, Tel Aviv, Israel, provided by Hortech systems Ltd.). Each system was also fitted with a Dosatron D3GL-2 feed injector (Tresses, France) used to provide plants with Kristalon Red NPK fertiliser (Yara, Grimsby, UK) between 24 March and 22 May 2014. This was set to mix fertiliser from a stock solution (made up at 100 g fertiliser per litre water) into water at a ratio of one part stock solution to 100 parts water before being sent to the pots. Plants were covered by 380 × 900 mm micro-perforated pollination bags (Focus Packaging & Design Ltd, Brigg, UK) once inflorescences began to show to prevent cross pollination. Any side shoots that emerged after bagging were cut. Watering was reduced to 50 % from 2 June 2014 and switched off completely from 1 July 2014 to encourage senescence. All plants were sprayed with 0.1 % (v/v) azoxystrobin (Amistar, Syngenta, Cambridge, UK) to control first signs of Phoma and some Botrytis on 20 November 2013 and were sprayed again on 29 January 2014 and 17 February 2014. Tebuconazole (Folicur, Bayer, Cambridge, UK) and Amistar were applied at a rate of 0.06 % (v/v) on 28 April 2014. Aphid control was by 0.05 % (w/v) Pirimicarb (Aphox, Syngenta) on 20 May 2014 and 0.07 % (v/v) Deltamethrin (Decis, Bayer) on 2 June 2014.

The total quantity of experimental units was 2160. All plants were harvested from the polytunnels from 14 to 17 July 2014. Stems were cut just above the bottom of the micro-perforated bag containing the top of plants. Each bag was then tied up such that no plant material could escape. Labelled bags were placed into 1 m^3^ ventilated crates for storage prior to threshing. Crates containing plant material were transported to Elsoms Seeds (Spalding, Lincolnshire), where they were threshed for seed with an SRC single plant thresher (Nickerson Brothers Limited, Lincoln) and cleaned using a Selecta seed cleaner (Selecta Machinefabriek B.V., Enkhuizen, Netherlands). Thousand seed weight for each plant was measured using a Contador seed counter (Pfeuffer GmbH, Kitzingen, Germany). Total seed yield per plant are indicative data, since side-stems were removed where these grew outside of the bags.

#### Sampling, digestion and analysis of leaf samples

Leaves were sampled at the rosette stage (typically 6–8 true leaves showing) from 5 to 11 March 2014. A minimum of three fully expanded leaves were cut from the plant, weighed and photographed while fresh. Leaves from each plant were stored in separate labelled paper bags at -20 °C. All samples were freeze dried (CHRIST Alpha 2-4 LD freeze dryer; Martin Christ Gefriertrocknungsanlagen GmbH, Osterode, Germany) for 48–60 h, and re-weighed. Leaves were homogenised in liquid N_2_ using a pestle and mortar and kept frozen prior to analyses.

Subsamples (~0.20 g DW) of leaf were digested using a microwave system comprising a Multiwave 3000 platform with a 48-vessel MF50 rotor (Anton Paar GmbH, Graz, Austria); digestion vessels were perfluoroalkoxy (PFA) liner material and polyethylethylketone (PEEK) pressure jackets (Anton Paar GmbH). Leaf material was digested in 2 mL 70 % Trace Analysis Grade HNO_3_, 1 mL Milli-Q water (18.2 MΩ cm; Fisher Scientific UK Ltd, Loughborough, UK), and 1 mL H_2_O_2_ with microwave settings as follows: power = 1400 W, temp = 140 °C, pressure = 2 MPa, time = 45 min. Two operational blanks were included in each digestion run. Duplicate samples of certified reference material (CRM) of leaf (Tomato SRM 1573a, NIST, Gaithersburg, MD, USA) were included approximately every fourth digestion run; laboratory reference material (LRM) from pooled / freeze-dried *Brassica napus* leaves was also used for later digests. Following digestion, each tube was made up to a final volume of 15 mL by adding 11 mL Milli-Q water and transferred to a 25 mL universal tube (Sarstedt Ltd., Nümbrecht, Germany) and stored at room temperature.

Leaf digestates were diluted 1-in-5 using Milli-Q water prior to elemental analysis. The concentrations of 28 elements were obtained using inductively coupled plasma-mass spectrometry (ICP-MS; Thermo Fisher Scientific iCAPQ, Thermo Fisher Scientific, Bremen, Germany); Ag, Al, As, B, Ba, Ca, Cd, Cr, Co, Cs, Cu, Fe, K, Mg, Mn, Mo, Na, Ni, P, Pb, Rb, S, Se, Sr, Ti, U, V, Zn. Operational modes included: (i) a helium collision-cell (He-cell) with kinetic energy discrimination to remove polyatomic interferences, (ii) standard mode (STD) in which the collision cell was evacuated, and (iii) a hydrogen collision-cell (H_2_-cell). Samples were introduced from an autosampler incorporating an ASXpress™ rapid uptake module (Cetac ASX-520, Teledyne Technologies Inc., Omaha, NE, USA) through a PEEK nebulizer (Burgener Mira Mist, Mississauga, Burgener Research Inc., Canada). Internal standards were introduced to the sample stream on a separate line via the ASXpress unit and included Sc (20 μg L^−1^), Rh (10 μg L^−1^), Ge (10 μg L^−1^) and Ir (5 μg L^−1^) in 2 % trace analysis grade HNO_3_ (Fisher Scientific UK Ltd). External multi-element calibration standards (Claritas-PPT grade CLMS-2; SPEX Certiprep Inc., Metuchen, NJ, USA) included Ag, Al, As, B, Ba, Cd, Ca, Co, Cr, Cs, Cu, Fe, K, Mg, Mn, Mo, Na, Ni, P, Pb, Rb, S, Se, Sr, Ti (semi-quant), U, V and Zn, in the range 0–100 μg L^−1^ (0, 20, 40, 100 μg L^−1^). A bespoke external multi-element calibration solution (PlasmaCAL, SCP Science, Courtaboeuf, France) was used to create Ca, K, Mg and Na standards in the range 0–30 mg L^−1^. Boron, P and S calibration utilized in-house standard solutions (KH_2_PO_4_, K_2_SO_4_ and H_3_BO_3_). In-sample switching was used to measure B and P in STD mode, Se in H_2_-cell mode and all other elements in He-cell mode. Sample processing was undertaken using Qtegra™ software (Thermo Fisher Scientific) with external cross-calibration between pulse-counting and analogue detector modes when required. In total, 2096 samples were analysed in 14 ICP-MS runs.

#### Digestion and analysis of seed samples

Dry seeds (three seeds per tube and occasionally four for very small seeds) were transferred into Pyrex test tubes (16 × 100 mm). After weighing an appropriate number of samples the masses of the remaining samples were calculated using method of Danku et al. [44]. The Seed samples were left overnight to pre-digest in 1.16 mL trace metal grade HNO_3_ (J. T. Baker Instra-Analyzed; Avantor Performance Materials; Scientific & Chemical Supplies Ltd, Aberdeen, UK) spiked with indium internal standard and 1.2 mL H_2_O_2_ (Primar-Trace analysis grade, 30 %; Fisher Scientific, Loughborough, UK) was also added. They were then digested in dry block heaters (DigiPREP MS, SCP Science; QMX Laboratories, Essex, UK) at 115 °C for 4 h.

Seed digestates were diluted to 11.5 mL with Milli-Q water (18.2 MΩ cm, Merck Millipore, Watford, UK) and aliquots transferred to 96-well deep well plates using adjustable multichannel pipette (Rainin; Anachem Ltd, Luton, UK) for analysis. Elemental analysis was performed with an ICP-MS (PerkinElmer NexION 300D equipped with Elemental Scientific Inc. autosampler and Apex HF sample introduction system; PerkinElmer LAS Ltd, Seer Green, UK and Elemental Scientific Inc., Omaha, NE, USA, respectively) in the standard mode. Twenty elements (Li, B, Na, Mg, P, S, K, Ca, Mn, Fe, Co, Ni, Cu, Zn, As, Se, Rb, Sr, Mo, and Cd) were monitored. Liquid reference material composed of pooled samples of the digested seed materials was prepared before the first sample run and was used throughout the remaining sample runs. The liquid reference material was included after every ninth sample in all ICP-MS sample sets to correct for variation between and within ICP-MS analysis runs [44]. Sample concentrations were calculated using external calibration method within the instrument software. The calibration standards (with indium internal standard and blanks) were prepared from single element standards (Inorganic Ventures; Essex Scientific Laboratory Supplies Ltd, Essex, UK) solutions. In total, 1986 samples were analysed across four ICP-MS runs.

#### Data processing of leaf and seed mineral composition traits

For each data-point, an element-specific operational blank concentration (mean of each ICP-MS run) was subtracted. Data were then multiplied by initial sample volume, divided by the initial dry mass of plant material, and converted to mg element kg^−1^ of dry leaf or seed material. Element-specific limits of detection (LODs) were reported as three times the standard deviation (SD) of the operational blank concentrations, assuming a notional starting dry weight of 0.200 g for leaf and 0.015 g for seed data (Additional file 1: Table S3). For leaves, element-specific recoveries from CRMs ranged from 68 to 134 %, for 18 elements with certified CRM values (Additional file 1: Table S4). From leaf mineral concentration data, seven elements (Ag, Co, Cr, Ni, Pb, U, V) were removed from further analysis due to having mean mineral concentrations which were less than or close to the LOD (Additional file 1: Table S5). Likewise, seven elements (As, Co, Cr, Fe, Ni, Pb, Se) were removed from seed mineral concentration data (Additional file 1: Table S6). For those elements retained for analysis, data for individual leaf and seed element concentrations which were below element-specific LODs were replaced with half LOD values. Leaf and seed element concentrations >5 standard deviation (SDs) above the global arithmetic mean for each element were also removed as a precaution against using contaminated samples (125 out of 58,688 values for leaves; 107 out of 42,504 values for seed).

The relative contribution of genotypic and non-genotypic variance components underlying variation in leaf and seed composition traits was calculated using a REML procedure in GenStat. Genotype, habit and experimental sources of variation were classed as random factors according to the model [habit + genotype + polytunnel + polytunnel/replicate + polytunnel/replicate/sub-block]. For leaf composition traits, a further model was used [replicate + (replicate/sub-block) + genotype + (replicate/genotype)] in which genotype was subsequently added as a fixed factor to estimate genotype-means. For seeds, the arithmetic mean data were used for each genotype.

### Multivariate analysis of root morphology and mineral composition traits

Correlation analysis was conducted on all 945 possible pairwise combinations of the 44 root, leaf and seed trait variate sets (genotype means). Five stepwise discriminant analyses were conducted in GenStat, one each for the root morphology-, leaf- and seed mineral- and seed weight variate sets, which contained 6, 21,15 and two variates, respectively, and one for the variate set of all traits combined. Genotypes were grouped according to ‘crop habit’. The Wilks’ Lambda ‘forward selection’ stepwise algorithm option was selected, which, at each step, adds the trait-variate which explains the most between-group variation from all of the remaining trait-variate sets. Specificity plots were drawn, to view the proportion of genotypes of each ‘crop habit’ correctly allocated to each group, at each step. Discrimination plots were drawn to represent the separation of variation in the crop habits in two dimensions. All statistical analyses were conducted using GenStat 15^th^ Edition (VSN International Ltd, Hemel Hempstead, UK).

## Results and discussion

### Root growth was influenced strongly by seed size

**Fig. 1:**
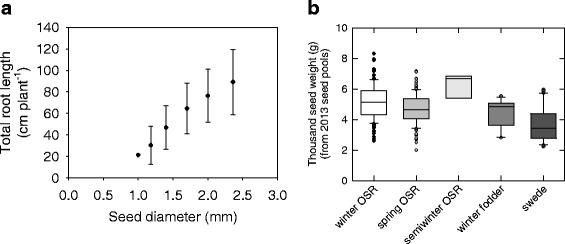
**a** Total root length (TRL) as a function of *Brassica napus* seed diameter from a ‘pouch and wick’ system. Data represent mean ± standard error of the mean for all seedlings grown, from seeds with diameters of 1.18, 1.40, 1.70, 2.0 and 2.36 mm; *n* = 44, 1349, 2055, 2242 and 1059, respectively, averaged across 361 genotypes. **b** Thousand seed weight (TSW) of the *B. napus* seed used in all experiments in this study. Data are means of up to 320 genotypes, including winter OSR (*n* = 142), spring OSR (*n* = 124), semiwinter OSR (*n* = 7), winter fodder (*n* = 14) and swede (*n* = 33) habits. Boxes represent the mid two quartiles with the median drawn; whiskers are the 95 % confidence limits plus extremes

Seed diameter accounted for a large proportion of the variation in total root length (TRL; 44 %), primary root length (PRL; 35 %), lateral root length (LRL; 41 %) and lateral root number (LRN; 41 %), but not for mean lateral root length (MLRL; 6 %) or lateral root density (LRD; 3 %) in 14 d old seedlings (Table 1; Fig. 1a). Genotype/habit factors accounted for between 7 % (MLRL) and 17 % (PRL) of the total variation in the six root traits. Residual (plant-to-plant) variation accounted for the largest single source of variation in the study, up to 75 and 81 % for LRD and MLRL, respectively, indicating that lateral roots traits are particularly responsive to the environment. This large residual source of variation is consistent with previous studies of *Brassica* seedling root traits, which show that large numbers of individuals are required to detect subtle differences in root traits between genotypes with confidence [5, 45]. Thousand seed weight (TSW) in the 2013 seed, from which all plants were grown, varied significantly within and between crop habits, from largest to smallest in: semiwinter OSR, winter OSR, spring OSR, winter fodder and swede types (*P* < 0.001, Fig. 1b). However, whilst seed diameter had a significant positive correlation with root length, based on the data for individual seedlings, potential correlations between TSW and root length could not be tested in this study because seeds were selected for uniformity between genotypes based on diameter classification and not by individual seed weights.

**Table 1:** Variance components analysis of root morphology, seed yield and leaf and seed mineral composition traits in *Brassica napus*, showing the variation (%) in the trait associated with genotype, habit, experimental design and residual factors, (seed size effect was calculated for the root traits only), as determined by Residual Maximum Likelihood (REML) analyses

Variate	Genotype	Habit	Experimental	Seed diameter	Residual
Root traits					
TRL	9	1	4	44	41
PRL	11	6	3	35	45
LRL	9	1	5	41	44
MLRL	6	1	6	5	81
LRN	9	4	5	41	43
LRD	13	2	8	3	75
Seed yield					
TSW	8	3	3	-	87
Leaf mineral composition					
Al	3	0	44	-	53
As	4	2	67	-	27
B	22	3	42	-	34
Ba	23	1	42	-	34
Ca	26	12	27	-	34
Cd	11	1	59	-	29
Cs	2	1	74	-	23
Cu	17	5	29	-	49
Fe	13	6	43	-	38
K	35	4	30	-	32
Mg	36	17	14	-	34
Mn	15	5	48	-	32
Mo	22	10	6	-	61
Na	37	21	6	-	36
P	21	8	17	-	55
Rb	30	7	35	-	28
S	40	15	17	-	28
Se	0	0	85	-	15
Sr	24	7	41	-	28
Ti	9	3	65	-	23
Zn	24	6	30	-	40
Seed mineral composition					
B	13	2	40	-	45
Ca	16	9	26	-	49
Cd	9	8	40	-	42
Cu	32	0	6	-	62
K	21	1	25	-	53
Li	10	5	16	-	69
Mg	6	18	52	-	24
Mn	12	1	55	-	32
Mo	41	21	7	-	31
Na	12	3	26	-	59
P	12	5	50	-	32
Rb	14	3	36	-	46
S	31	13	37	-	19
Sr	9	8	27	-	57
Zn	21	12	14	-	53

*TRL* total root length, *PRL* primary root length, *LRL* total lateral root length, *MLRL* mean lateral root length, *LRN* lateral root number, *LRD* lateral root density, *TSW* Thousand Seed Weight. See Additional file 1: Table S10 for detailed information

Positive relationships have been reported previously between seed size and the seminal root length and total root weight of barley (*Hordeum vulgare*) [46], and between seed size and total root size and lateral root number in tomato (*Solanum lycopersicum*) [47]. Larger seeds have also been shown to improve seedling establishment, shoot weight, biomass and final yield in some, but not all field studies of OSR in Canada [48–51]. However, larger-sized seeds typically had more vigorous early growth [51]. Thousand seed weight was also shown to correlate positively with absolute growth rate 21 days after germination [52]. Improved seed size-related root growth of *B. napus* seedlings might also increase tolerance to shoot pests (e.g. flea beetle, *Phyllotreta* spp.) [48] and root diseases such as *Rhizoctonia solani* which can damage the primary roots of *B. napus* [53]. Seed weight has previously been associated with pre-emergence growth in a bi-parental mapping population of *Brassica oleracea*, but under separate genetic control to germination [54, 55]. Additionally, the present study found that the thousand seed weight (TSW) in the winter OSR varieties from different release periods has steadily increased over time, suggesting that larger seeds may have been bred for (Additional file 2: Figure S6). This present study shows there is scope to exploit the genetic control of seed size-related root growth as a potential route to improve early vigour in the small-seeded *B. napus*.

### Winter OSR and fodder types had larger root systems than other crop habits

**Fig. 2:**
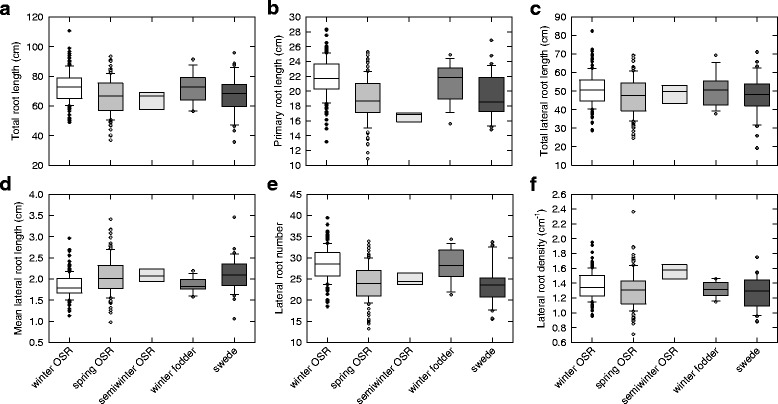
Root traits of *Brassica napus* grown in a ‘pouch and wick’ system. Data are means of up to 319 genotypes, including winter OSR (*n* = 142), spring OSR (*n* = 124), semiwinter OSR (*n* = 7), winter fodder (*n* = 14) and swede (*n* = 32) habits. Boxes represent the mid two quartiles with the median drawn; whiskers are the 95 % confidence limits plus extremes. Panels **a**–**f** represent different root traits

Winter OSR and winter fodder types had a greater mean TRL, PRL, TLRL, and LNR at 14 d than the other crop habits (*P* < 0.001, Fig. 2; Additional file 1: Table S7). Semiwinter OSR had a shorter mean PRL than all other habits (*P* < 0.001, Fig. 2b), and a greater mean LRD (*P* < 0.001, Fig. 2f). It is important to note that these differences in root system size between OSR crop types were observed when seeds of uniform diameter were sown for each genotype. Increased root length in seedlings is likely to indicate increased early vigour. Velicka et al. [56] observed that early sowing afforded a greater root collar thickness and leaf number, and these earlier sown plants had greater over-winter survival and more rapid accumulation of matter in the apical bud in spring. Furthermore, Scott et al. [57] observed that earlier sowing significantly increased seed yield because of increased leaf and root growth. Seedling root-length traits, measured in this same ‘pouch and wick’ system, correlated with early plant growth and final seed yield in 30 commercial winter OSR *B. napus* genotypes [5]. Finch-Savage et al. [55] suggested that vigorous early root growth is essential for small seeded crops such as *Brassica* to acquire resources before desiccation occurs. A fast-growing, thick root collar contains large amounts of soluble carbohydrates which will enable the plant to withstand frost and afford a rapid re-growth in spring [58]. Thus, sufficient early root growth is necessary for winter crop survival and may have been selected for based on yield in previous breeding programs, whereas spring sown crops have less need for a rapid development to ensure hardiness.

### Spring varieties typically had higher leaf concentrations of macronutrients and some micronutrients than winter varieties

**Fig. 3:**
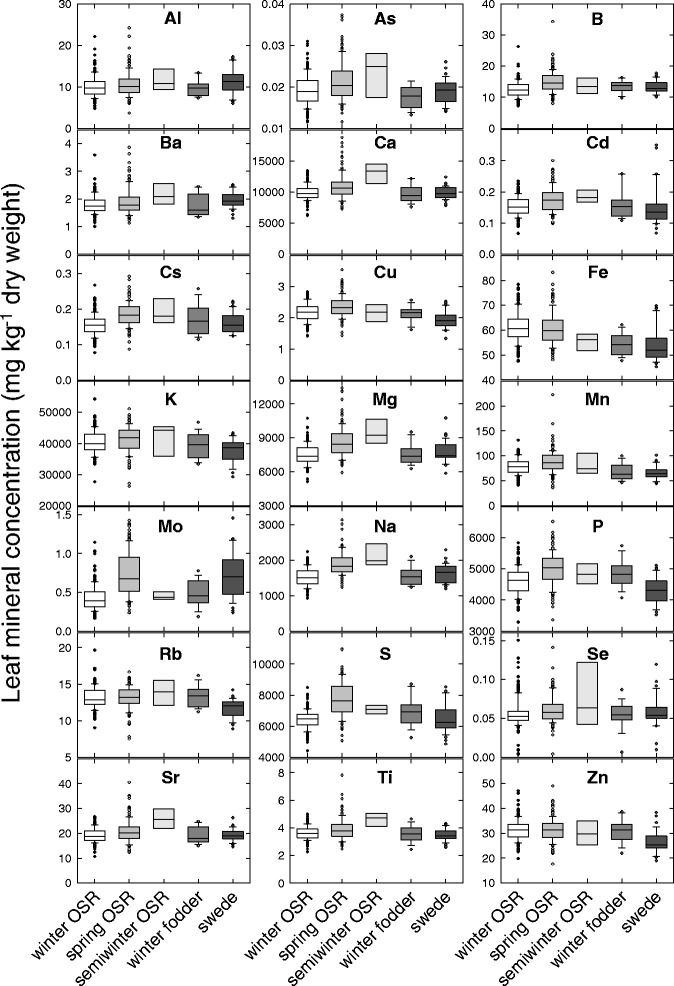
Leaf mineral concentrations of *Brassica napus* grown in compost. Data are means of up to 385 genotypes, including winter OSR (*n* = 163), spring OSR (*n* = 127), semiwinter OSR (*n* = 7), winter fodder (*n* = 15) and swede (*n* = 35) habits. Boxes represent the mid two quartiles with the median drawn; whiskers are the 95 % confidence limits plus extremes

The mean leaf concentration of 21 elements varied by more than six orders of magnitude across genotypes, from 0.01 mg kg^−1^ (As) to >50,000 mg kg^−1^ (K) (Fig. 3; Additional file 1: Table S7). Genotypic variation in leaf mineral concentration ranged from 1.8-fold (Fe) to >40-fold (Se). Among the macronutrients, leaf mineral concentrations varied 2.0-fold for P, 2.1-fold for K, 3.0-fold for Ca, 2.6-fold for Mg, and 2.5-fold variation in S. In comparison, among a panel of ~450 *B. oleracea,* also grown in compost and sampled during early vegetative growth, shoot concentrations of: Ca and Mg varied 2.0- and 2.3-fold [21], respectively; P and K varied 4.9- [59] and 3.4-fold [60], respectively. Among a panel of soil-grown 509 inbred lines of *B. napus*, the shoot mineral concentrations of 30 d old seedlings varied (approximately) 2.0-fold for Ca, 1.6-fold for Mg, 6.7-fold for P and 2.0-fold for K [23].

Winter OSR, winter fodder and swede had lower mean leaf macronutrient (Ca, Mg, P, K, S) concentrations than spring and semiwinter OSR (Ca, Mg; *P* < 0.001, Fig. 3). Semiwinter OSR also had higher mean leaf Ca and Mg concentrations compared to other habits (*P* < 0.001). Among the micronutrients, leaf Cu was greater in spring OSR than other habits (*P* < 0.001). Leaf Fe concentrations were greater in winter and spring OSR *(P* < 0.001). The mean leaf Mo concentrations were greatest in Spring OSR and swede (*P* < 0.001), although there was substantial variation within crop type. The mean leaf concentrations of beneficial and non-essential elements (As, Cd, Na, Se) were consistently higher in the semiwinter OSR leaves used in this study, typically followed by spring OSR (Fig. 3). Likewise, in the study of Bus et al. [23], winter OSR also had lower mean shoot Ca, K and S concentrations than spring and semiwinter OSR, but similar P concentrations and semiwinter OSR had the highest shoot S and Zn of the crop types. Despite these overall trends in the data, there is wide variation in shoot mineral composition within all crop types of *B. napus*, which will be influenced strongly by the nutritional environment in which the plant is grown as well as genotypic factors.

Variance components analysis (Table 1; Additional file 1: Table S10) shows that genotype had the largest influence on leaf S concentration (40 %) and the smallest influence on leaf Se concentrations (0 %). Habit accounted for the least variation in all traits (generally less than 10 %) but had the greatest effect on leaf Na, Mg and S concentration. The trends of heritabilities (i.e. genotype effect) for leaf composition traits follow a similar pattern to those observed previously in soil grown leaves of *Arabidopsis* [61], whereby leaf Mg was the most heritable macro nutrient in their study, and the second most heritable in this study. Leaf Ca, K and Mo concentration were ranked among the most heritable leaf composition traits in both studies. Leaf Fe, Mn and Cu concentration were among the least heritable traits in both studies. The variance components analysis indicates that the effect of experimental variance is generally higher for micronutrients than macronutrients.

### Seed mineral concentrations were consistent across habits for many nutrients, but S concentrations were lower and Mo concentrations were higher in OSR types

**Fig. 4:**
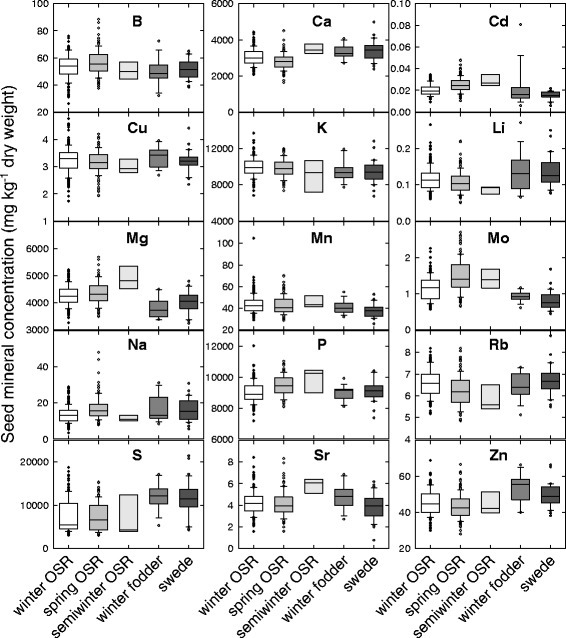
Seed mineral concentrations of *Brassica napus* grown in compost. Data are means of up to 380 genotypes, including winter OSR (*n* = 162), spring OSR (*n* = 127), semiwinter OSR (*n* = 7), winter fodder (*n* = 15) and swede (*n* = 31) habits. Boxes represent the mid two quartiles with the median drawn; whiskers are the 95 % confidence limits plus extremes

The mean seed concentration of 15 elements varied by more than six orders of magnitude across genotypes, from 0.01 mg kg^−1^ (Cd) to >13,000 mg kg^−1^ (K) (Fig. 4; Additional file 1: Table S7). Genotypic variation in seed mineral concentration varied 1.7-fold (P) to 14-fold (Na). Among the macronutrients, seed mineral concentrations varied 3.1-fold for Ca, 1.9-fold for Mg, 2.0-fold for K, and 7.5-fold for S. We are not aware of previous reports of species-wide variation in seed mineral composition traits in a *Brassica* species. White and Broadley [14] reviewed variation in the mineral composition of edible cereal grains and dicot seeds for several species, typically core germplasm collections, which had been grown under comparative conditions. Among the dicots, seed Ca concentration varied 3.7-, 2.0-, 9.1-, 1.5- and 1.9-fold, and seed Mg concentration varied 2.4-, 1.4-, 2.3-, 1.3-, and 1.6-fold, for chickpea, peanut, pea, bean and soybean, respectively. Therefore, the seed macronutrient composition of this *B. napus* panel appears to be a similar range as other dicot species.

Winter and spring OSR had similar seed macronutrient concentrations, except for P, in which spring and semiwinter OSR had higher seed P concentrations than winter types (*P* < 0.001, Fig. 4) and Mg in which the semiwinter had higher concentrations than other types (*P* < 0.001). Winter fodder and swede had higher seed S concentrations than OSR crop types (*P* < 0.001), presumably due to the smaller proportion of “double-low” (low glucosinolate, low erucic acid) compared to “double-high” varieties, because winter fodder and swede have not been selected for low seed glucosinolate concentration. Glucosinolates are sulphur and nitrogen-containing secondary metabolites common in the *Brassicaceae* family [62, 63], and some genes of the sulphate assimilation pathway are members of the glucosinolate biosynthetic network [64]. Seed Mo concentrations showed the opposite pattern, with higher concentrations in OSR than other types (*P* < 0.001). An antagonistic effect of sulphate on Mo concentration in the shoot, root and seeds of OSR has previously been observed [65, 66]. Given that S and Mo are known to share some assimilation and transport pathways [67, 68], this could imply a relationship between seed glucosinolates, S and Mo. It has previously been suggested that tissue specific demand can regulate the expression of sulphate transporters [69]. It could be surmised that plants with low seed S/glucosinolates display a slightly different sulphate transporter profile leading to differences in Mo accumulation. Nevertheless, how S and Mo are interacting in relation to glucosinolates is yet to be determined. Consistent with S/Mo transport being under strong genetic control, variance components analysis (Table 1; Additional file 1: Table S10) showed that genotype had the largest influence on seed Mo concentration (41 %) out of all the elements analysed in this study. As observed previously in *Arabidopsis* seeds [61], Mo, Cu, S and K concentrations were influenced by genotype to a greater extent than seed Mg and P concentrations. Of the micronutrients, seed B, Cu and Mn concentrations were generally consistent across habits; B was highest in spring OSR (*P* < 0.001), Cu was highest in winter fodder and winter OSR (*P* < 0.001), Mn was highest in semiwinter OSR (*P* < 0.001). Zn concentrations were the highest in winter fodder (*P* < 0.001), Cd concentrations were highest in semiwinter OSR (*P* < 0.001), and seed Na concentrations were highest in spring OSR.

### Differences in nutrient translocation between crop habits can be detected by comparing leaf and seed concentration ratios of elements with potentially similar transport or assimilation pathways

**Fig. 5:**
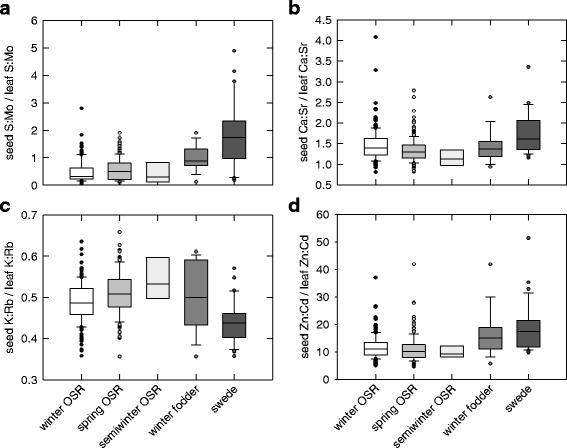
Leaf and seed element concentration ratios of *Brassica napus* grown in compost. Data are means of up to 385 and 380 genotypes, for leaf and seed concentrations, respectively, including winter OSR (*n* = 163), spring OSR (*n* = 127), semiwinter OSR (*n* = 7), winter fodder (*n* = 15) and swede (*n* = 35) habits. Boxes represent the mid two quartiles with the median drawn; whiskers are the 95 % confidence limits plus extremes. Ratios are calculated, e.g. for Ca:Sr, as ([S]_seed_/[Mo]_seed_) / ([S]_leaf_/[Mo]_leaf_). Panels **a**–**d** represent different element pairs

Four pairs of elements were selected, for which both leaf and seed concentration data were available, and for which there are reports of shared transport/assimilation pathways. These element pairs are S and Mo [67, 68], Ca and Sr [70, 71], K and Rb [72–74], and Zn and Cd [75]. The second element of each of these pairs is either not a nutrient, or, for Mo, is only required in very small amounts compared to the first element in each pair. The first hypothesis tested was that the ratio of first:second element concentration in leaves is ≫ 1:1, as expected from external nutrient supply and plant requirements. The second hypothesis was that the seed ratio/leaf ratio of elements is >1:1. For example, in the case of S and Mo, this would be expressed as ([S]_seed_/[Mo]_seed_) / ([S]_leaf_/[Mo]_leaf_; Fig. 5a). A seed ratio/leaf ratio of elements >1:1 indicates that the net seed accumulation of the essential, or more abundant, nutrient element, is greater than the non-essential, or less abundant, nutrient. This could be due to selective processes in the source (e.g. increased mobilisation from the leaf) and/or sink (e.g. decreased remobilisation from the pod) tissues. As expected, the ratio of first:second element is ≫ 1:1 in leaves. For S:Mo, the mean of 387 genotypes is 15902:1 (range 4808–37661:1); for Ca:Sr 519:1 (433–604:1); for K:Rb 3106:1 (2652–3760:1); for Zn:Cd 200:1 (85–449:1). However, there was considerable variation in the seed/leaf ratio of elements (Fig. 5; Additional file 1: Table S7). For S:Mo, the differences between habits was most apparent. For the OSR habits, most OSR genotypes had seed ratio/leaf ratios <1:1, indicating net accumulation of Mo in seeds is greater than S in relative terms (Fig. 5c). In contrast, most swede genotypes and almost half of the winter fodder genotypes had a greater net accumulation of S than Mo. This relationship is indicative of a potential link between glucosinolates, S and Mo content of seeds but the nature of the interactions between these components has yet to be elucidated and is the focus of current studies on this population. For Ca:Sr, most genotypes showed slight increase in net accumulation of Ca compared to Sr in seeds, with swede habits having the highest mean (Fig. 5b). Interestingly, all genotypes showed increased net accumulation of Rb in seeds compared to K, with swede habits showing the lowest relative net accumulation of K compared to Rb (Fig. 5c). The reasons for this observation are not clear. For Zn:Cd, all genotypes showed increased net Zn accumulation in seeds compared to Cd. This was again greater in swede habits, and also winter fodder; both had greater net accumulation of Zn in seeds, compared to Cd, than did the OSR habits.

### Root traits and leaf and seed mineral composition traits correlate within, but not between tissues

**Fig. 6:**
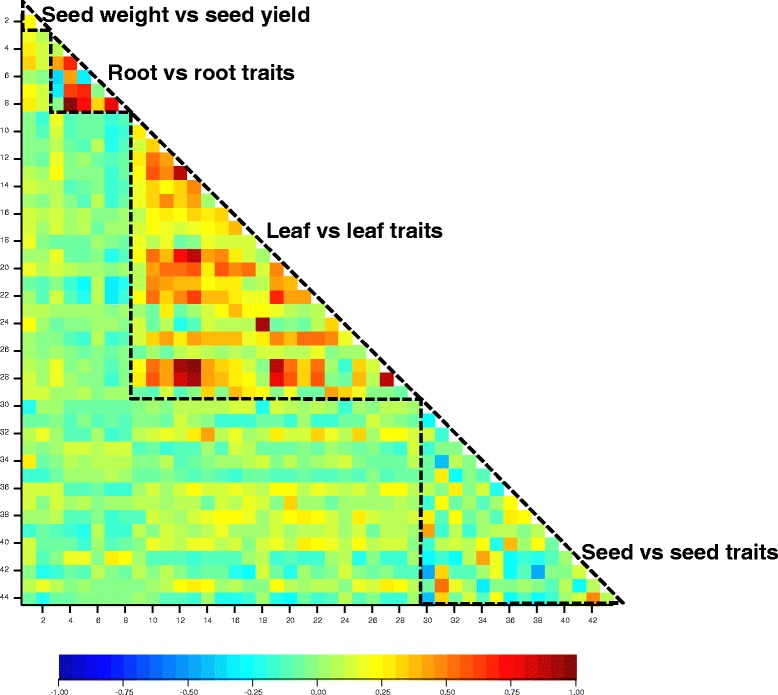
Pair-wise correlations of all 44 traits; TSW and seed yield, root morphology and leaf and seed mineral composition traits in *Brassica napus* genotypes. Plants were grown in a ‘pouch and wick’ system (root traits, up to 319 genotypes) or compost (leaf and seed traits; up to 385 and 380 genotypes, respectively). Correlation coefficients are scaled from -1.0 (*dark blue*) to +1.0 (*dark red*) and are from genotype trait means. Traits: (1) TSW; (2) seed yield; (3–8) root traits: (3) LRD; (4) LRL; (5) LRN; (6) MLRL; (7) PRL; (8) TRL; (9–29) leaf concentration traits: (9) Al; (10) As; (11) B; (12) Ba; (13) Ca; (14) Cd; (15) Cs; (16) Cu; (17) Fe; (18) K; (19) Mg; (20) Mn; (21) Mo; (22) Na; (23) P; (24) Rb; (25) S; (26) Se; (27) Sr; (28) Ti; (29) Zn; (30–44) seed concentration traits: (30) B; (31) Ca; (32) Cd; (33) Cu; (34) K; (35) Li; (36) Mg; (37) Mn; (38) Mo; (39) Na; (40) P; (41) Rb; (42) S; (43) Sr; (44) Zn

There were strong correlations between root traits. The strongest positive correlations were between traits relating directly to the total length of the root system (i.e. comprising PRL and LRL components, *P* < 0.001) and also between these traits and LRN (*P* < 0.001, Fig. 6; Additional file 1: Table S8). There was a weak negative relationship between PRL and LRD (*r* = -0.28; *P* < *0.001*), since the latter is derived from LRN/PRL. In addition, there was also a weak negative relationship between MLRL and LRD (*r* = -0.38; *P* < *0.001*), suggesting a trade-off between lateral root length and number. These correlations are consistent with previous observations on a much smaller panel, of 32 UK-field adapted OSR genotypes, grown in the same ‘pouch and wick’ system [5]. There were many strong positive correlations between the leaf concentrations of pairs of elements (Fig. 6; Additional file 1: Table S8). The strongest positive relationships were between leaf concentrations of Ca and Sr (*r* = 0.97), Sr and Ba (*r* = 0.93), K and Rb (*r* = 0.92) and Ca and Mg (*r* = 0.87); all *P* < *0.001*. Positive correlations between the leaf concentrations of Group II elements reflect the relative lack of selectivity between these elements during transport within the plant [70]. Such relationships between Ca and Mg have been observed previously within-species, including among panels of diverse *B. oleracea* [21], OSR [23], Arabidopsis [61] and multi-species datasets [17, 73]. The strong positive relationship in the leaf concentration of Group I elements K and Rb is as expected from previous observation across many species [74]. However, the transport of other Group I elements is typically much more selective than Group II elements [71]. There were few negative relationships between the leaf concentrations of pairs of elements (Fig. 6), but weak negative relationships were observed between Rb and the Group II elements, Ba (*r* = -0.24), Sr (*r* = -0.18), and Ca (*r* = -0.16); all *P* < *0.001*.

There were few strong positive correlations between the seed concentrations of pairs of elements (Fig. 6; Additional file 1: Table S8). The strongest positive correlation was between Ca and Sr (*r* = 0.51; *P* < *0.001*), followed by between K and Rb (*r* = 0.40; *P* < *0.001*). Both of these correlations were much weaker than those observed in leaves. The strongest negative correlations between the seed concentrations of pairs of elements were between S and Mo (*r* = -0.47), S and B (*r* = -0.46), and Ca and K (*r* = -0.40); all *P* < *0.001* (Fig. 6).

In general, leaf and seed mineral composition traits correlated very weakly (Fig. 6; Additional file 1: Table S8). The strongest positive correlation in a compositional trait between plant parts was a positive correlation between leaf Cd and seed Cd (*r* = 0.41; *P* < *0.001*). All other correlations between leaf and seed mineral composition traits, and between root traits and leaf or seed composition traits were weaker, with correlation coefficients ranging from -0.26 to +0.33. Likewise, with the exception of P, no correlations were observed between elemental concentrations of leaf, root and seed tissues in *Arabidopsis* [61].

The strongest positive correlations between a seedling root trait and a leaf composition trait were between LRD and leaf Ca (*r* = 0.16; *P* = 0.006), Sr (*r* = 0.15; *P* = 0.008) and Ba (*r* = 0.14; *P* = 0.01) concentrations. A similar weak positive correlation between seedling LRD and leaf Ca (and Zn) concentrations was also seen previously in some field experiments [5]. Interestingly, PRL had weak, but significant, negative correlations with most leaf composition traits e.g. Mo (*r* = 0.31, *P* = 0.001) and Na (*r* = 0.30, *P* = 0.001). These data provide some evidence that LRD might be a beneficial trait for nutrient resource and above-ground biomass acquisition in *B. napus* [5]. Some leaf and seed mineral concentrations e.g. Ca and Mg and some beneficial elements (Fig. 3) of semiwinter OSR genotypes were greater than in other habits (Fig. 2f). Semiwinter OSR genotypes used as starting material for this study had the greatest LRD and greatest mean TSW of the five crop habits, which may have led to improved overall root size and function, and mineral acquisition in this study (Additional file 2: Figure S5). Semiwinter types are likely to have a distinct breeding history from winter and spring OSR habits due to having more introgressions from *B. rapa* and a longer period of domestication [76, 77]. These differences in pedigree might also explain some of the variation in root and shoot traits assigned in this present study to crop habit, and warrant further study in a range of environments.

### Combining root, leaf and seed traits in a discriminant analysis is characteristic of crop habit

**Fig. 7:**
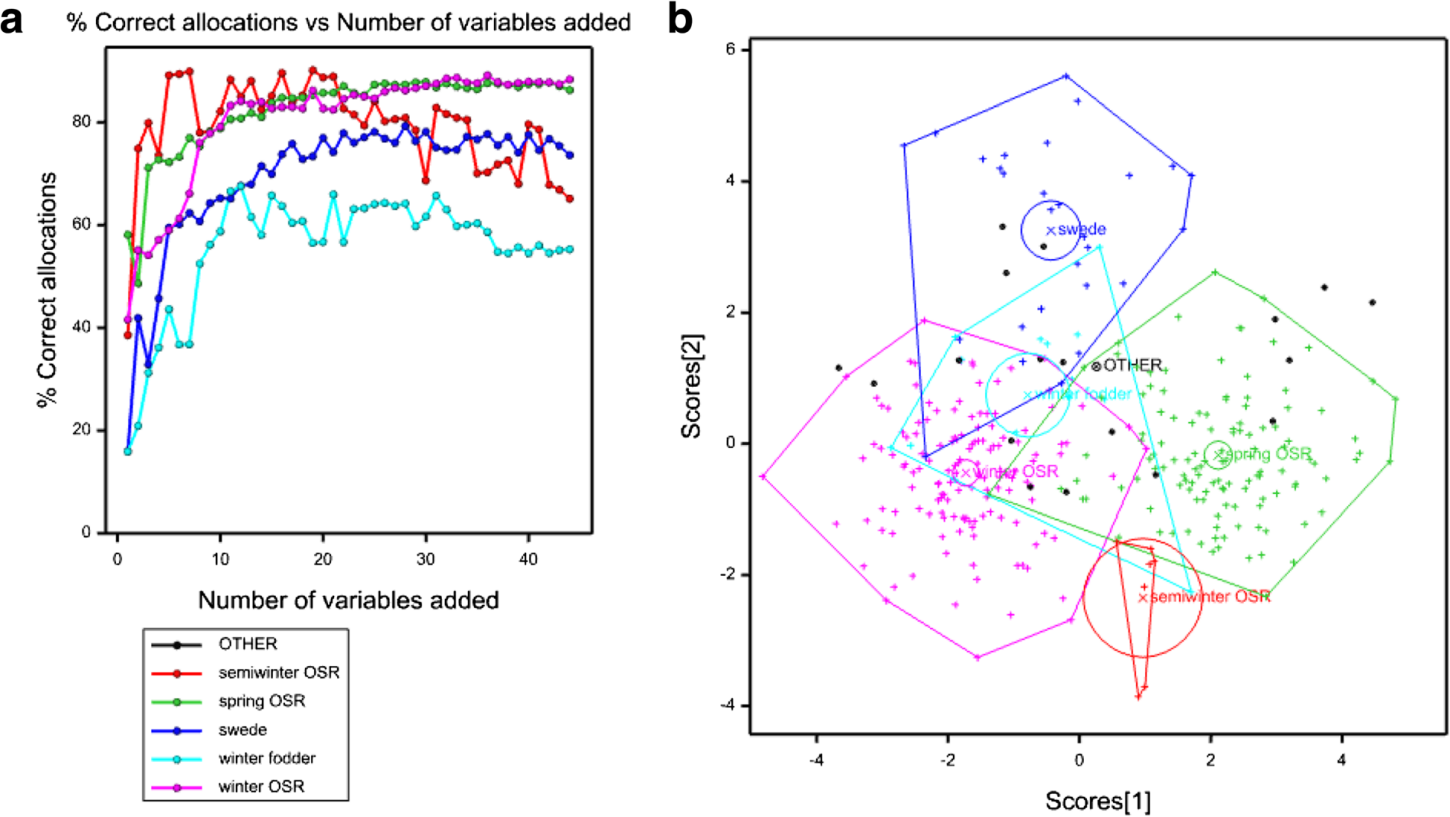
Step-wise discriminant analysis using the variate set of all 44 traits; root morphology, TSW and seed yield and leaf and seed mineral composition traits combined. **a** Specificity plot showing the proportion of genotypes of each ‘crop habit’ correctly allocated to each group, at each step of the discriminant analysis; (**b**) discrimination plots drawn to represent the distribution of ‘crop habits’ in two dimensional variance space. ‘Crop habit’ means (X), 95 % confidence circles (*circles*) and group polygons enclosing all units for each crop habit are indicated

Combining all traits within the discriminant analysis provided the most accurate characterisation of crop habit (Fig. 7). Using the variate set of all traits combined, and after the addition of the 20 most informative trait variate sets, genotypes with the winter (86 %), spring (85 %) and semiwinter (81 %) OSR habits were correctly allocated to the correct group, as were 76 % of swede and 68 % of the winter fodder habits. The trait which contributed the most in terms of allocation to crop habit was leaf S concentration, followed by PRL, and seed Ca and Mo concentration, followed by the TSW of the 2013 seed from which plants were grown. The next most important root trait after PRL was MLRL, which was ranked 12^th^. The relative contributions of all 44 traits in this variate set to the discriminant analysis are presented in Additional file 1: Table S9. The discriminant analyses for each of the root-, leaf- and seed-trait variate sets was less accurate (Additional file 2: Figures S1–S4).

## Conclusions

*Brassica napus* has been shown to be amenable to rapid marker identification linked to useful agronomic traits in wide diversity populations. For example, using associative transcriptomics (AT) with a panel of 84 *B. napus* genotypes from within the same panel used here, Harper et al. [37] showed that seed contents of both erucic acid and glucosinolates (GS) were associated with specific genes known to be involved in their biosynthetic pathways, whilst identifying additional new target loci of potential use in breeding. Similarly, Koprivova et al. [78] used a subset of this study’s population to identify novel loci linked to shoot anion accumulation. The AT technique is based on transcriptome-sequencing, combined with association mapping. It uses transcribed sequences (mRNA-seq) which allows variation of gene sequences to be detected (through single nucleotide polymorphisms; SNPs) whilst reducing the complexity of the analysis compared to typical genome-wide association analysis (GWAS). In addition, transcript abundance (gene expression markers; GEMs) can be quantified simultaneously. Transcript abundance is likely to be of particular relevance in the control of traits in complex polyploid species in which gene duplication may lead to unequal expression of gene paralogues [79].

A substantial proportion of the same inbred population used in this study have also been used to study other traits, including those relating to nutritional composition and seedling growth, in other environments [23, 38–40, 80] and those based on leaf ionome traits [25–31]. The most accurate characterisation of crop habit is when multiple plant part traits are combined in analysis. Thus, combining multiple datasets using this panel has the potential for more accurate candidate trait identification, and to dissect the transport pathways which lead to altered elemental accumulation for crop improvement. The volume and throughput of data obtained from root phenotyping and ionomics platforms is considerable and the challenge now is to combine datasets for a better understanding of the ionome and the key traits involved in elemental accumulation, and to mine for the underlying molecular mechanisms of these useful traits using associative transcriptomics analysis.

## Additional files

Additional file 1: Supplementary tables. A collection of tables containing a variety of extra data including raw data gathered in the experiments, limits of detection calculations, data used for analyses of traits, and data used for generation of some of the figures. Also included is a detailed ouput from variance components analysis and a list of the abbreviations used across the tables. (XLSX 1324 kb)
Additional file 2: Supplementary figures. A collection of extra figures which may be of interest to readers but that aren’t in the main scope of the submission. Figures S1–S4 show step-wise discriminant analyses plots using different subsets of the traits measured (root morphology traits, leaf mineral composition traits, seed mineral composition traits, & seed yield traits respectively). Plots from analyses using the full set of traits are included as a main figure in the submission; see Fig. 7. Figures S5 and S6 are box and whisker plots of seed yield data by crop habit and thousand seed weight by genotype release date respectively. (PPTX 429 kb)

## Abbreviations

Ag: Silver
Al: Aluminium
As: Arsenic
B: Boron
Ba: Barium
Ca: Calcium
Cd: Cadmium
Co: Cobalt
Cr: Chromium
Cs: Caesium
Cu: Copper
Fe: Iron
K: Potassium
LRD: Lateral root density
LRL: Lateral root length
LRN: Lateral root number
Mg: Magnesium
MLRL: Mean lateral root length
Mn: Manganese
Mo: Molybdenum
Na: Sodium
Ni: Nickel
P: Phosphorus
Pb: Lead
PRL: Primary root length
Rb: Rubidium
S: Sulphur
Se: Selenium
Sr: Strontium
Ti: Titanium
TRL: Total root length
TSW: Thousand seed weight
U: Uranium
V: Vanadium
Zn: Zinc
