# Supplementary material for: Root morphology and seed and leaf ionomic traits in a Brassica napus L. diversity panel show wide phenotypic variation and are characteristic of crop habit
Source: BMC Plant Biol. 2016 Oct 4;16:214. doi: 10.1186/s12870-016-0902-5 (PMC5050600; doi:10.1186/s12870-016-0902-5)
Supplement: Additional file 2: — Supplementary figures. A collection of extra figures which may be of interest to readers but that aren’t in the main scope of the submission. Figures S1–S4 show step-wise discriminant analyses plots using different subsets of the traits measured (root morphology traits, leaf mineral composition traits, seed mineral composition traits, & seed yield traits respectively). Plots from analyses using the full set of traits are included as a main figure in the submission; see Fig. 7. Figures S5 and S6 are box and whisker plots of seed yield data by crop habit and thousand seed weight by genotype release date respectively. (PPTX 429 kb) [file 12870_2016_902_MOESM2_ESM.pptx]

## Slide 1
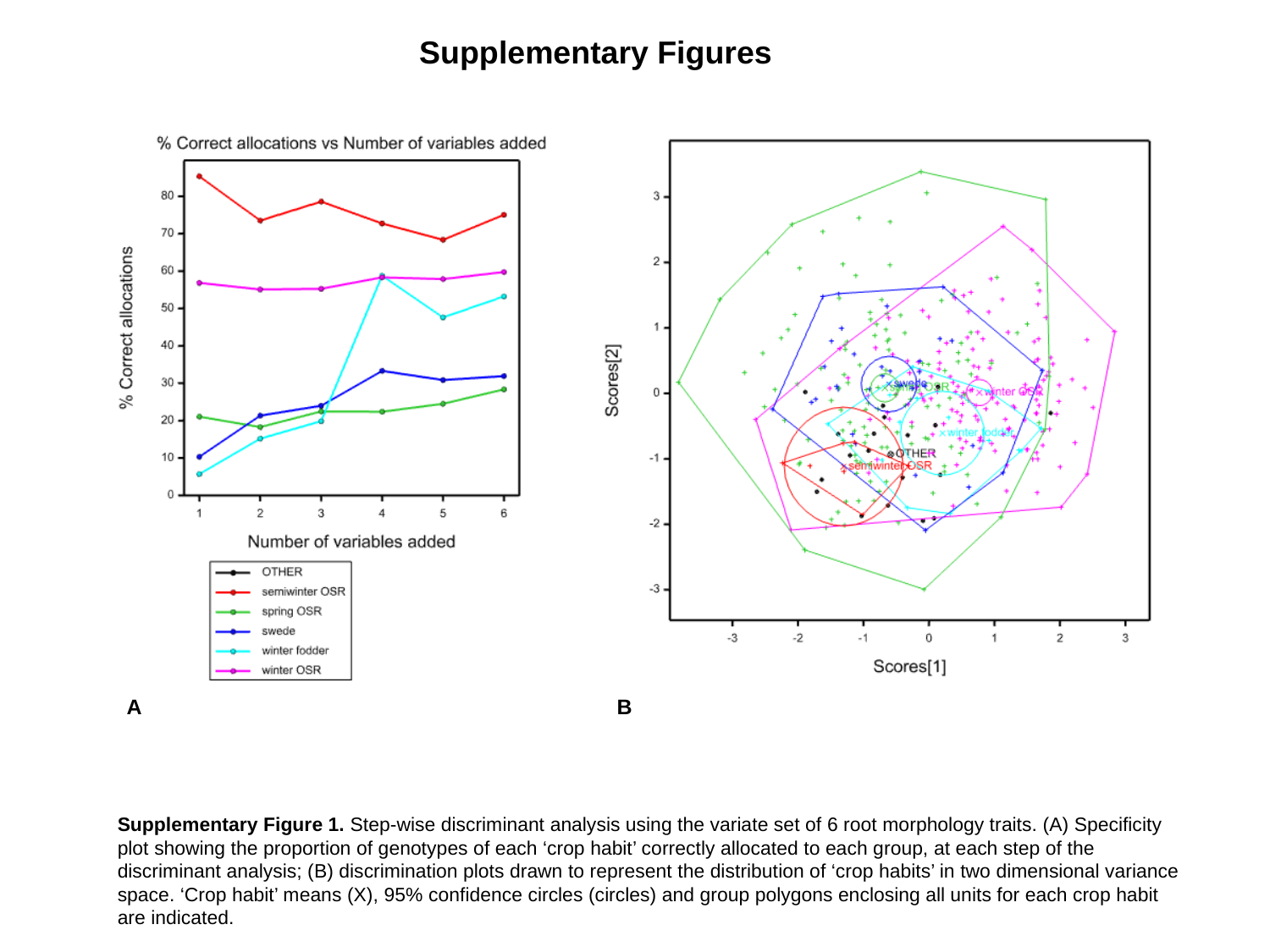

Supplementary Figures
A
B
Supplementary Figure 1. Step-wise discriminant analysis using the variate set of 6 root morphology traits. (A) Specificity plot showing the proportion of genotypes of each ‘crop habit’ correctly allocated to each group, at each step of the discriminant analysis; (B) discrimination plots drawn to represent the distribution of ‘crop habits’ in two dimensional variance space. ‘Crop habit’ means (X), 95% confidence circles (circles) and group polygons enclosing all units for each crop habit are indicated.

## Slide 2
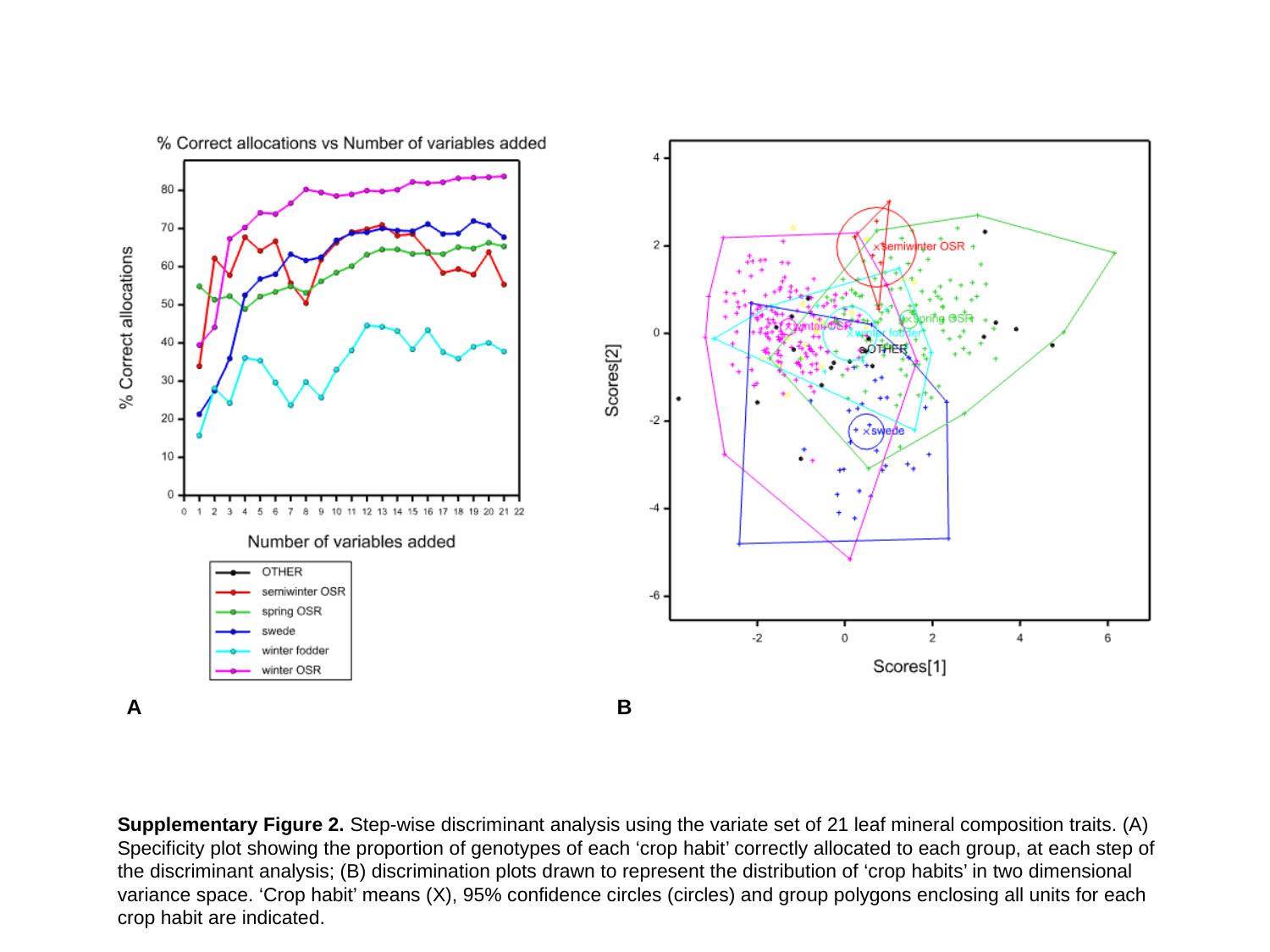

A
B
Supplementary Figure 2. Step-wise discriminant analysis using the variate set of 21 leaf mineral composition traits. (A) Specificity plot showing the proportion of genotypes of each ‘crop habit’ correctly allocated to each group, at each step of the discriminant analysis; (B) discrimination plots drawn to represent the distribution of ‘crop habits’ in two dimensional variance space. ‘Crop habit’ means (X), 95% confidence circles (circles) and group polygons enclosing all units for each crop habit are indicated.

## Slide 3
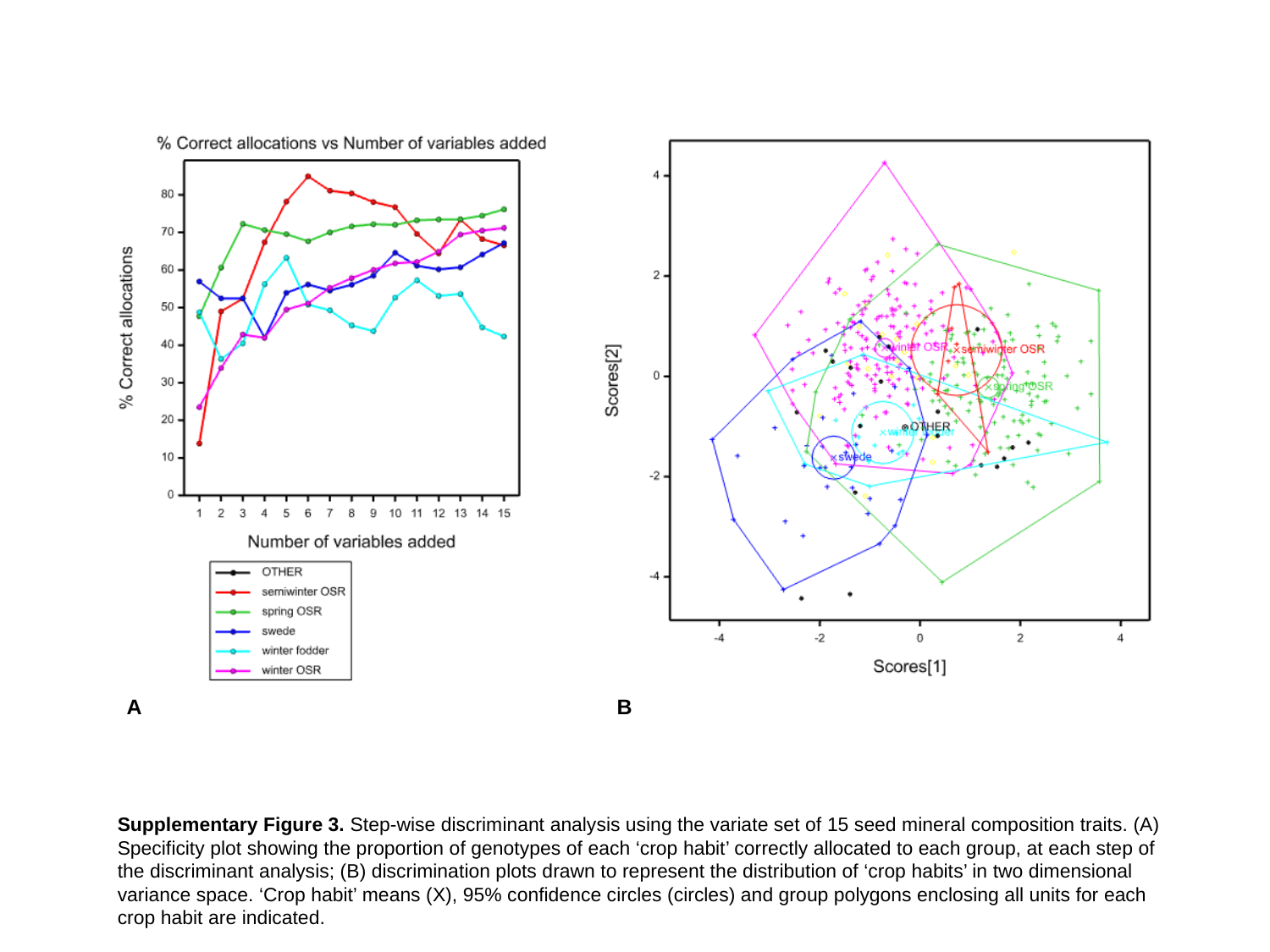

A
B
Supplementary Figure 3. Step-wise discriminant analysis using the variate set of 15 seed mineral composition traits. (A) Specificity plot showing the proportion of genotypes of each ‘crop habit’ correctly allocated to each group, at each step of the discriminant analysis; (B) discrimination plots drawn to represent the distribution of ‘crop habits’ in two dimensional variance space. ‘Crop habit’ means (X), 95% confidence circles (circles) and group polygons enclosing all units for each crop habit are indicated.

## Slide 4
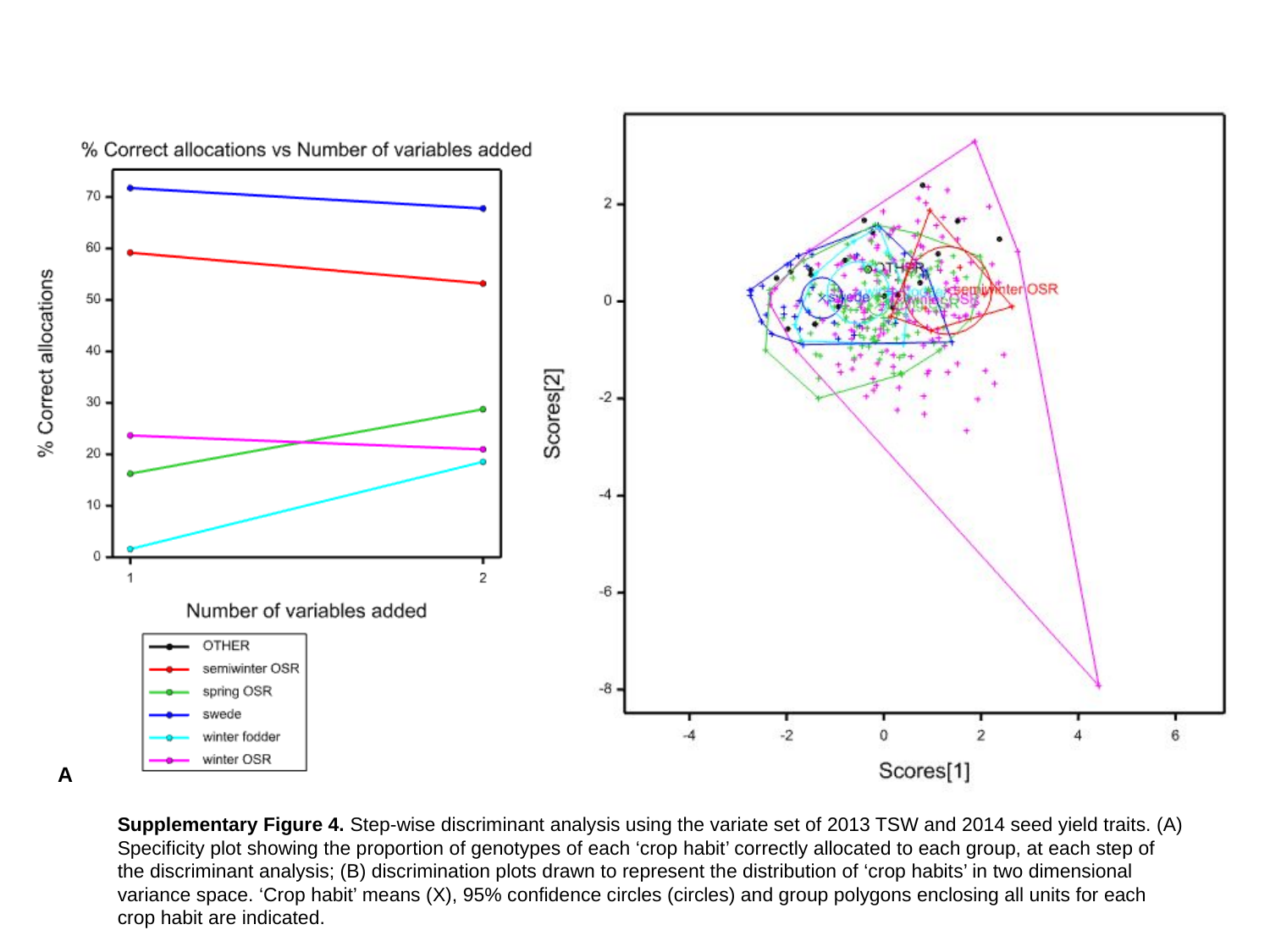

B
A
Supplementary Figure 4. Step-wise discriminant analysis using the variate set of 2013 TSW and 2014 seed yield traits. (A) Specificity plot showing the proportion of genotypes of each ‘crop habit’ correctly allocated to each group, at each step of the discriminant analysis; (B) discrimination plots drawn to represent the distribution of ‘crop habits’ in two dimensional variance space. ‘Crop habit’ means (X), 95% confidence circles (circles) and group polygons enclosing all units for each crop habit are indicated.

## Slide 5
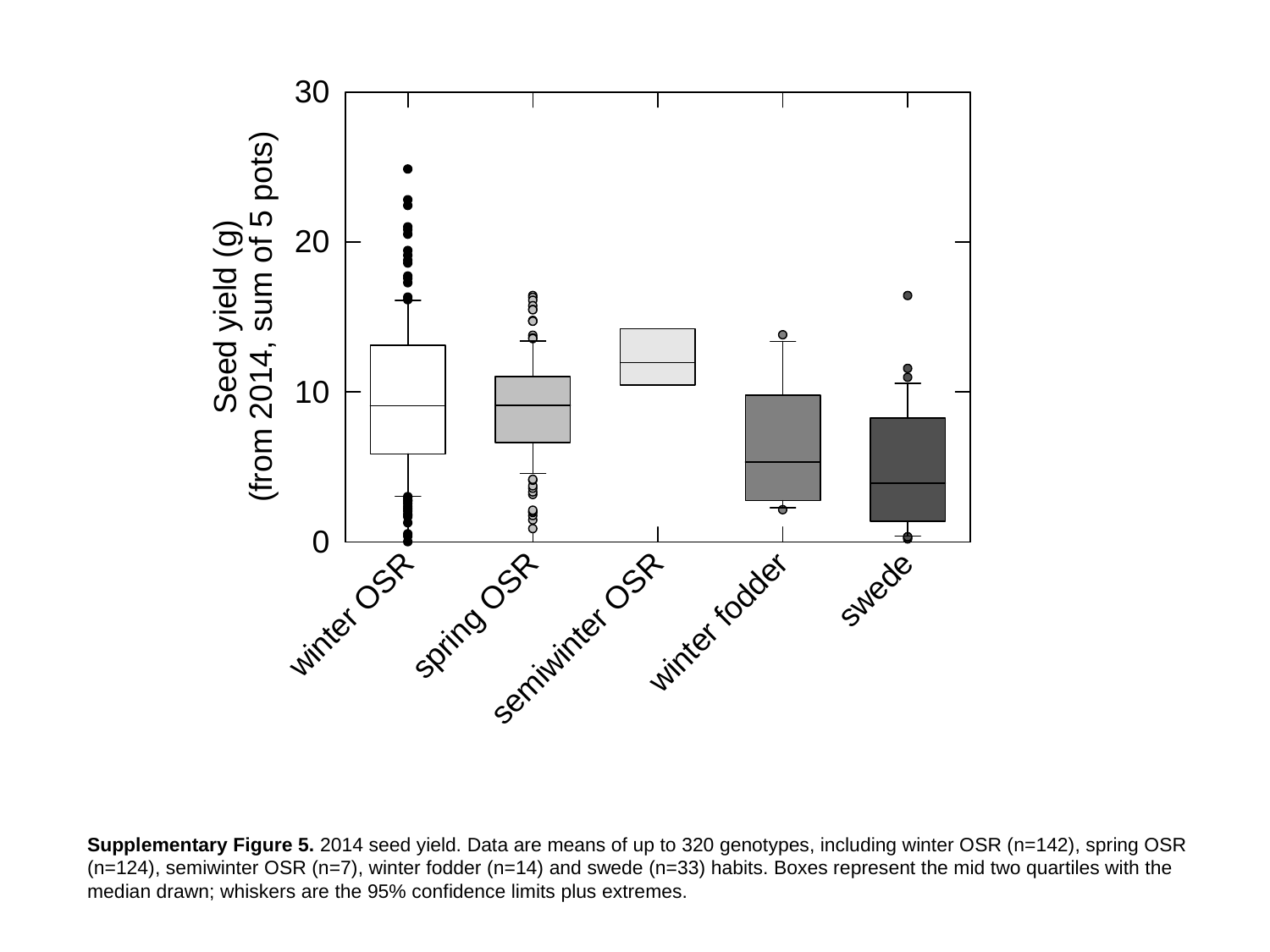

Supplementary Figure 5. 2014 seed yield. Data are means of up to 320 genotypes, including winter OSR (n=142), spring OSR (n=124), semiwinter OSR (n=7), winter fodder (n=14) and swede (n=33) habits. Boxes represent the mid two quartiles with the median drawn; whiskers are the 95% confidence limits plus extremes.

## Slide 6
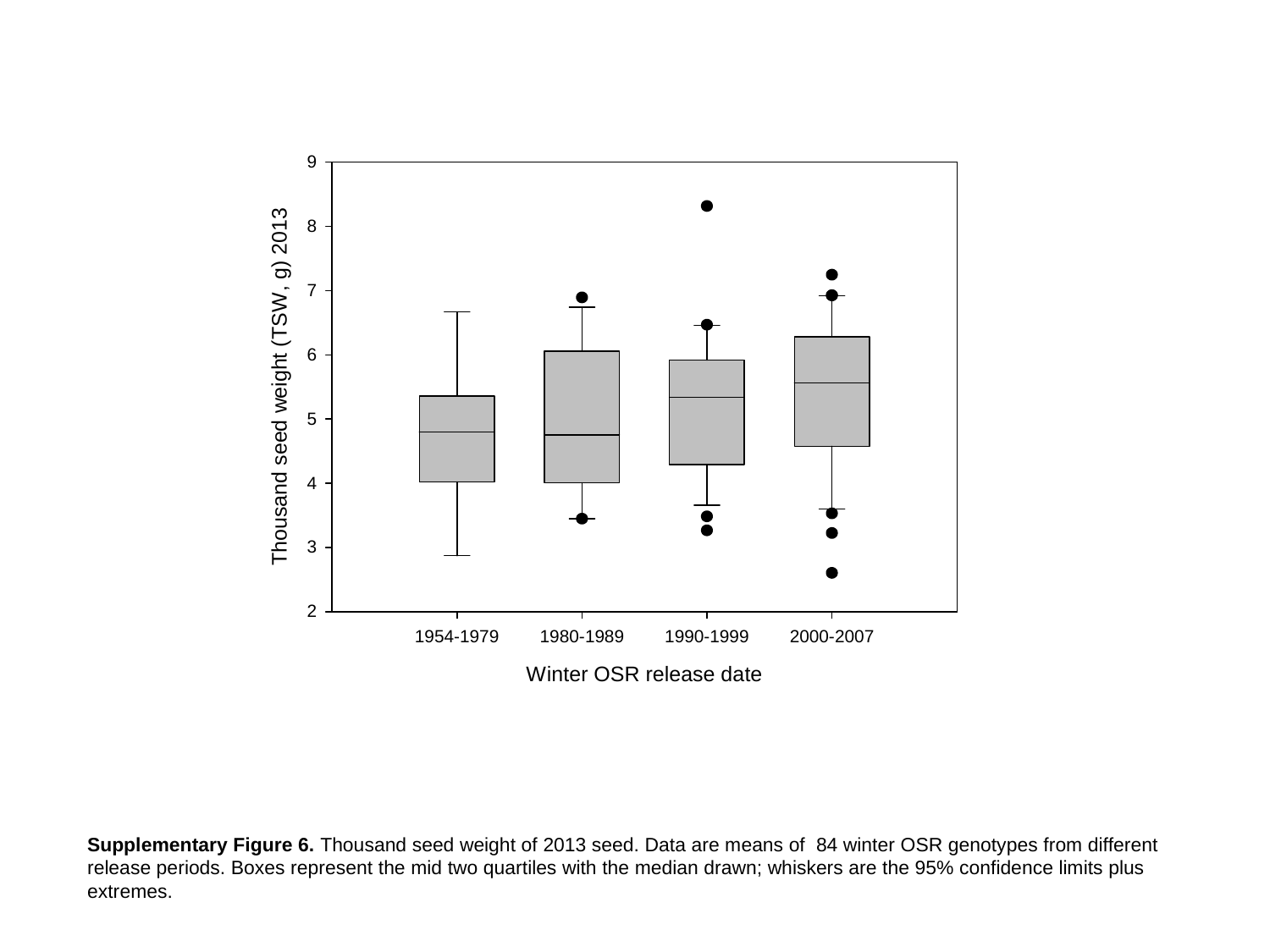

Supplementary Figure 6. Thousand seed weight of 2013 seed. Data are means of 84 winter OSR genotypes from different release periods. Boxes represent the mid two quartiles with the median drawn; whiskers are the 95% confidence limits plus extremes.
